# miRNA-146b Targets *TRAF6* and Inhibits LTA-Induced Inflammation of Bovine Mammary Epithelial Cells

**DOI:** 10.3390/ani16060958

**Published:** 2026-03-19

**Authors:** Yangyang Song, Peng Liu, Mingxue Li, Xiaolin Li, Huaxue Song, Yutong Zhang, Fanzhi Kong, Changyuan Wang, Binglei Shen

**Affiliations:** College of Animal Science and Technology, Heilongjiang Bayi Agricultural University, Daqing 163319, China; songyangyang@byau.edu.cn (Y.S.); 18346663533@163.com (P.L.); 13847560653@163.com (M.L.); lxl13795131329@163.com (X.L.); 13359929549@163.com (H.S.); 13136755125@163.com (Y.Z.); fanzhikong110@hotmail.com (F.K.); byndwcy@163.com (C.W.)

**Keywords:** miR-146b, mastitis, MAC-T cells, inflammation

## Abstract

Mastitis is an inflammatory disease of mammary tissue that impairs milk production and quality, thereby seriously threatening the economic viability of dairy farms. miRNAs, such as miR-146b, are emerging as new candidates for anti-inflammatory therapy, and their activity modulation may provide a basis for controlling inflammation. However, the exact role and underlying mechanisms of miR-146b in bovine mastitis and associated inflammatory pathways in bovine mammary epithelial cells are still unclear. To clarify this, we established an in vitro model by stimulating MAC-T cells with Lipoteichoic acid and investigated the miR-146b-associated molecular pathways. The data from this study showed increased miR-146b expression after LTA stimulation. Then, the dual-luciferase reporter assay identified *TRAF6* as a genuine target of miR-146b. Overexpression of miR-146b repressed the *TRAF6* level and NF-κB pathway activation. Consequently, the production and secretion of major pro-inflammatory cytokines, including TNF-α, IL-1β, and IL-6, were significantly repressed, which could be indicative of inhibition of the inflammation in the cell model. Meanwhile, knockdown of miR-146b abolished such benefits. Based on these findings, it is reasonable to conclude that miR-146b mitigates LTA-induced inflammatory responses in bovine mammary epithelial cells by directly targeting *TRAF6* and downstream NF-κB signaling. The present study identifies the functional role of miR-146b in the regulation of inflammation in MAC-T cells and highlights its potential as a target in mastitis intervention.

## Simple Summary

The aim of this study was to investigate the regulatory effect of miR-146b on lipoteichoic acid-induced inflammatory response in dairy cow mammary epithelial cells. Our results indicate that miR-146b targets *TRAF6*, inhibits inflammation in dairy cow mammary epithelial cells, and promotes their cell viability. miR-146b and its target gene *TRAF6* play a certain role in mammary gland development in dairy cows, which is expected to become an effective target for the treatment of mastitis.

## 1. Introduction

Bovine mastitis is an inflammatory disease of the mammary gland induced by physical, chemical, or microbial factors and is considered one of the most economically devastating diseases in the worldwide dairy industry [1,2]. Subclinical mastitis is difficult to diagnose because of the absence of apparent symptoms. *Staphylococcus aureus* is known to be one of the predominant pathogens causing subclinical mastitis in dairy cattle [3]. One of the major virulence components of this pathogen is Lipoteichoic acid (LTA), which strongly provokes the secretion of various inflammatory factors (TNF-α, IL-6, and IL-1β) from host cells [4]. Thus, the inhibition of subsequent inflammatory signaling pathways provides a potential approach for alleviating mammary glands damage.

Immune balance within the mammary gland is maintained by coordinated interactions among bovine mammary epithelial cells (MAC-T), lymphocytes, macrophages, and neutrophils present in the tissue [5]. Besides their primary role in milk production, the MAC-T cells form an important barrier against pathogenic invasion and play a central role in udder immune defense [6]. These cells participate in the early recognition of pathogens and initiate innate immune reactions, thus organizing further immune responses at both the cellular and molecular levels [7]. A number of studies have shown that MAC-T in culture are able to sense bacteria or bacterial products and respond by upregulating several sets of genes involved in the innate immune response [8]. MAC-T cell is widely regarded as a reliable in vitro model for studies of mammary gland functions because of its expression of milk-specific proteins [9]. While antibiotics remain the main treatment for mastitis, their use is associated with negative consequences such as drug residues and antimicrobial resistance [10]. For these reasons, developing alternative non-antibiotic treatments for bovine mastitis is considered an urgent priority.

MicroRNA is one class of non-coding small RNA molecules, approximately 18–22 nucleotides in length, that play an important role in regulating several pathological processes [11]. The evidence accumulated thus far has demonstrated that miRNAs may be secreted and subsequently taken into specific effector cells to exert functional regulation, mainly by modulating post-transcriptional gene expression [12]. Among these, miR-146b was implicated as a key regulator in immune responses, inflammatory processes, and cytokine signal transduction. Lei Zhang et al. (2020) found that MIR-146b prevented inflammatory damage through the MyD88/NF-κB signaling pathway in pediatric pneumonia [13]. Shuying He et al. (2025) found that miR-146b alleviated inflammatory bowel disease (IBD) by inducing anti-inflammatory effects of IL-10 reprogrammed macrophage polarization [14]. Further studies by Chou Lou et al. (2023) showed that miR-146b-5p inhibited inflammatory responses by targeting *TRAF6* [15]. S. Matis et al. (2022) documented that miR-146b-5p targets the IL-23 receptor and its downstream signaling components in the process of regulation of immune responses and cell survival [16]. Ruoxi He et al. (2019) found that overexpression of miR-146b in murine alveolar macrophages reduced LPS-induced TNF-α and IL-1β release [17]. These results suggest that miR-146b plays an important role in immune regulation and inflammation.

Tumor necrosis factor receptor-associated factor 6 (*TRAF6*), a member of the TRAF family with E3 ubiquitin ligase activity [18], acts as a pivotal node in activating various inflammatory signaling pathways [19,20]. In addition to its established functions in innate and adaptive immune responses, *TRAF6* is important in processes such as embryonic development and tissue homeostasis [18]. Until now, the relationship between miR-146b and *TRAF6* in the pathogenesis of bovine mastitis has not been reported. Based on these previous studies, we hypothesized that miR-146b targets *TRAF6* to exert anti-inflammatory effects in MAC-T cells. In this study, LTA-induced dairy cow mammary epithelial cells were used to explore the effect of miR-146b on inflammation in dairy cow mammary epithelial cells, and to provide a new direction for analyzing the molecular regulatory network and finding molecular therapeutic targets for dairy cow mastitis.

## 2. Materials and Methods

### 2.1. Cell Culture and Treatment

MAC-T and HEK293T cell were both obtained from Heilongjiang Bayi Agricultural University. MAC-T and HEK293T cell lines were cultured in Dulbecco’s Modified Eagle Medium (c11995500, Gibco, Grand Island, NY, USA) supplemented with 10% fetal bovine serum (164210, Pricella, Wuhan, China). The cells were incubated at 37 °C in a humidified atmosphere of 5% CO_2_. MAC-T cells were cultured to 80% cell density and then stimulated with 20 μg/mL LTA for 0, 6, 8, 12, and 24 h to determine the optimal stimulation time for the LTA model. The dose of 20 μg/mL LTA can stimulate MAC-T cells [21,22].

### 2.2. Cell Transfection

For transfection experiments, cells were seeded in 6-well plates at a density of 2.0 × 10^5^ cells per well. The cells were transfected with either a miR-146b mimic or inhibitor (Songon, Shanghai, China) using an appropriate mRNA transfection reagent, following the manufacturer’s instructions. The experimental design included the following groups: negative control (NC), LTA-induced inflammation in MAC-T cells (LTA), mimic NC, miR-146b mimic, inhibitor NC, and miR-146b inhibitor.

### 2.3. Transcription-Quantitative Polymerase Chain Reaction (RT-qPCR)

Extraction of total RNA was carried out using TRIzol reagent. Reverse transcription of miRNAs was performed with the PrimeScript™ RT reagent Kit with gDNA Eraser (RR047Q, Takara, Beijing, China) in accordance with the manufacturer’s protocol. Amplification was achieved by qPCR on a CFX96 RT-PCR detection system utilizing a TB Green Premix Ex Taq^TM^ (RR420Q, TaKaRa, China). The expression levels of miR-146b, *TRAF6*, *TNF-α*, *IL-1β*, *IL-6*, *U6*, and *GAPDH* (Sangon, Shanghai, China) were quantified. The sequences of the primers employed are presented in Table 1. Analysis of relative expression was conducted using the comparative Ct (2^−ΔΔCt^) method.

### 2.4. Western Blot

Protein samples (10 μL) were separated by SDS-PAGE. Subsequently, the separated proteins were electrophoresed and transferred to a polyvinylidene difluoride (PVDF) membrane. Blocked with 5% skimmed milk for 2 h. After washing three times with TBST for 15 min, the membranes were incubated with primary antibodies, including anti-TRAF6 antibody (1:1000, A16991, ABclonal, Wuhan, China), anti-TNF-α antibody (1:1000, EPR19147, Abcam, Cambridge, UK), anti-IL-6 antibody (1:1000, EPR21711, Abcam, Waltham, MA, USA), Anti-il-1β antibody (1:1000, EPR16805-15, Abcam, USA), anti-GAPDH antibody (1:50,000, 1E6D9, China) were incubated at 4 °C for 12 h. After incubation, the membranes were washed three times with tris-buffered saline (TBST) containing Tween-20 for 10 min. The membranes were incubated with HRP Conjugated AffiniPure Goat Anti-rabbit IgG (H + L) (1:10,000, BA1054, Boster, Wuhan, China) for 60 min and washed with TBST for another 10 min for three times. Finally, the cells were immersed in enhanced chemiluminescence (ECL) substrates. Protein bands were visualized using a Bio-Rad ChemiDoc imaging system (Bio-Rad, Hercules, CA, USA), and images were quantified using ImageJ software 1.8.0.

### 2.5. CCK-8 Cells Viability Assay

MAC-T cells were plated in 96-well plates and allowed to adhere for 24 h. Subsequently, the cells were treated with specific stimuli as required by the experimental protocol. After a 48 h incubation period, 10 μL of CCK-8 (MA0218, MeilunBio, Dalian, China) solution was added to each well. Finally, following a 2 h incubation at 37 °C, the absorbance was recorded at 450 nm.

*Cell viability* = [(*As* − *Ab*)/(*Ac* − *Ab*)] × 100%

As: absorbance of experimental wells (medium containing cells, CCK-8, drug to be tested).

Ac: absorbance of control wells (medium containing cells, CCK-8, and no drug under test).

Ab: absorbance of blank wells (medium without cells and drug to be tested, CCK-8).

### 2.6. Dual-Luciferase Reporter Gene Assay

HEK293T cells were seeded into 96-well plates at a density of 1 × 10^4^ cells per well and cultured under standard conditions (37 °C, 5% CO_2_, saturated humidity). The wild-type (WT) and mutant (MUT) 3′UTR sequences of *TRAF6* mRNA, along with a negative control (NC) sequence, were cloned into a reporter vector. According to the manufacturer’s protocol, the recombinant vectors were co-transfected with either miR-146b mimic or mimic NC into the cells using Lipofectamine 3000 (BL623, Biosharp, Beijing, China). After 6 h of incubation, the culture medium was replaced with fresh complete medium. At 48 h post-transfection, firefly and Renilla luciferase activities were measured using a dual-luciferase reporter assay kit (RG088, Beyotime, Shanghai, China), following the manufacturer’s instructions. All experiments were performed in triplicate.

### 2.7. Statistical Analysis of Data

Data are presented as mean ± standard deviation (SD) from at least three independent experiments. The normality of data distribution was assessed with the Shapiro–Wilk test. Statistical analysis was performed using GraphPad Prism 8.0. A two-tailed Student’s *t*-test was used for two-group comparisons. Multiple group comparisons were conducted using one-way ANOVA followed by Tukey’s post hoc test. Statistical significance was defined as a *p*-value ≤ 0.05.

## 3. Results

### 3.1. Time-Dependent Screening for LTA-Induced MAC-T Inflammatory Model Establishment

**Figure 1:**
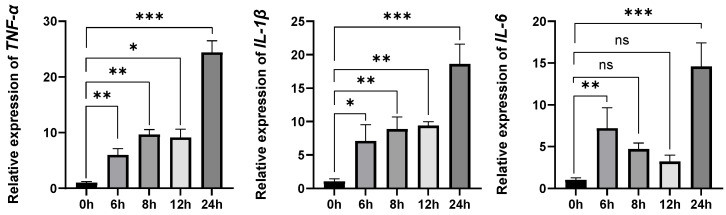
Effects of LTA stimulation on the expression of *TNF-α*, *IL-6* and *IL-1β* in MAC-T cells at different times. ns, not significant (*p* > 0.05), * refers to *p* < 0.05, ** refers to *p* < 0.01, *** refers to *p* < 0.001.

The qRT-PCR assay was used to detect the expression of inflammatory factors in MAC-T cells after stimulation with 20 μg/mL LTA at 0 h, 6 h, 8 h, 12 h, and 24 h. The results showed that after LTA stimulation of MAC for 24 h, the expressions of inflammatory factors *TNF-α*, *IL-6*, and *IL-1β* were significantly up-regulated (Figure 1).

### 3.2. Effect of LTA on the Expression of miR-146b and TRAF6 in MAC-T Cells

**Figure 2:**
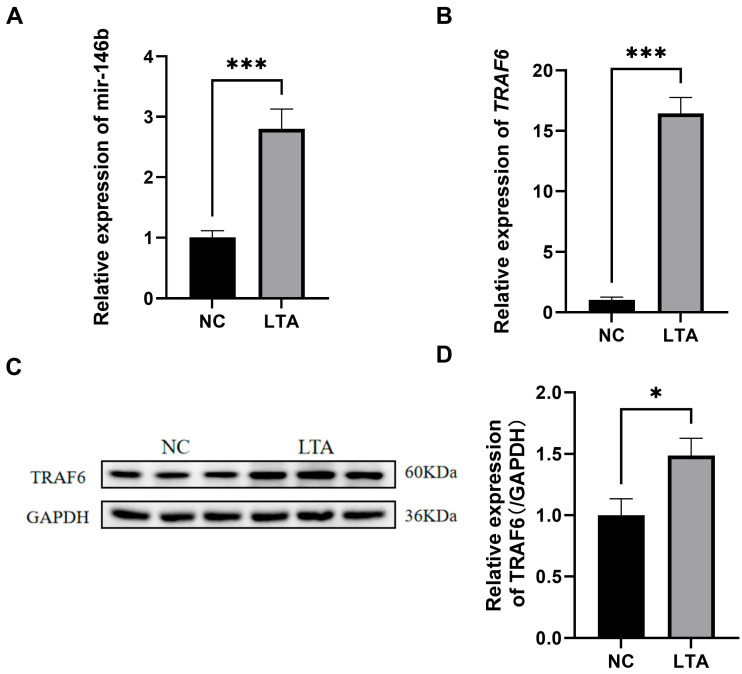
Effects of LTA on the expression of miR-146b and *TRAF6* in MAC-T cells. (**A**) mRNA expression level of miR-146b in MAC-T cells induced by LTA. (**B**) mRNA expression level of *TRAF6* in MAC-T cells induced by LTA. (**C**) Protein expression level of TRAF6 in MAC-T cells induced by LTA. (**D**) Relative expression of TRAF6 (/GAPDH). * refers to *p* < 0.05, *** refers to *p* < 0.001.

To further investigate the role of miR-146b in LTA-induced bovine mastitis, we first examined its expression in MAC-T cells following LTA stimulation. MAC-T cells were treated with LTA for 24 h to establish an inflammatory model. Compared with the control group, LTA challenge significantly up-regulated the expression of miR-146b (Figure 2A). Additionally, *TRAF6* mRNA levels were also increased (Figure 2B). Western blot analysis further revealed that TRAF6 protein expression was elevated in LTA-induced MAC-T cells relative to the NC group (Figure 2C).

### 3.3. Effect of miR-146b on the Viability of LTA-Induced MAC-T Cells

**Figure 3:**
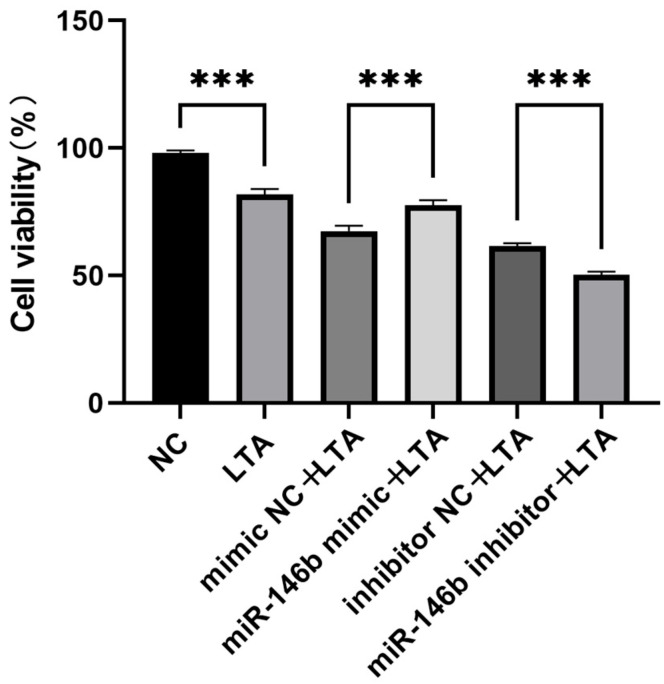
Effect of miR-146b on the viability of LTA-induced MAC-T cells. The impact of miR-146b mimic or inhibitor on the viability of LTA-induced MAC-T cells was assessed using the CCK-8 assay, and the results were normalized. *** refers to *p* < 0.001.

The effect of miR-146b on the viability of LTA-induced MAC-T cells was assessed using the CCK-8 assay. MAC-T cells were seeded in 96-well plates and treated with either an miR-146b mimic or an miR-146b inhibitor. The results showed that compared with the NC group, cell viability was significantly decreased in the LTA-treated group. In contrast, overexpression of miR-146b markedly increased cell viability relative to the mimic NC group, while inhibition of miR-146b led to a significant reduction in cell viability compared to the inhibitor NC group (Figure 3).

### 3.4. Effect of miR-146b on TRAF6 Expression in HEK293T Cells

**Figure 4:**
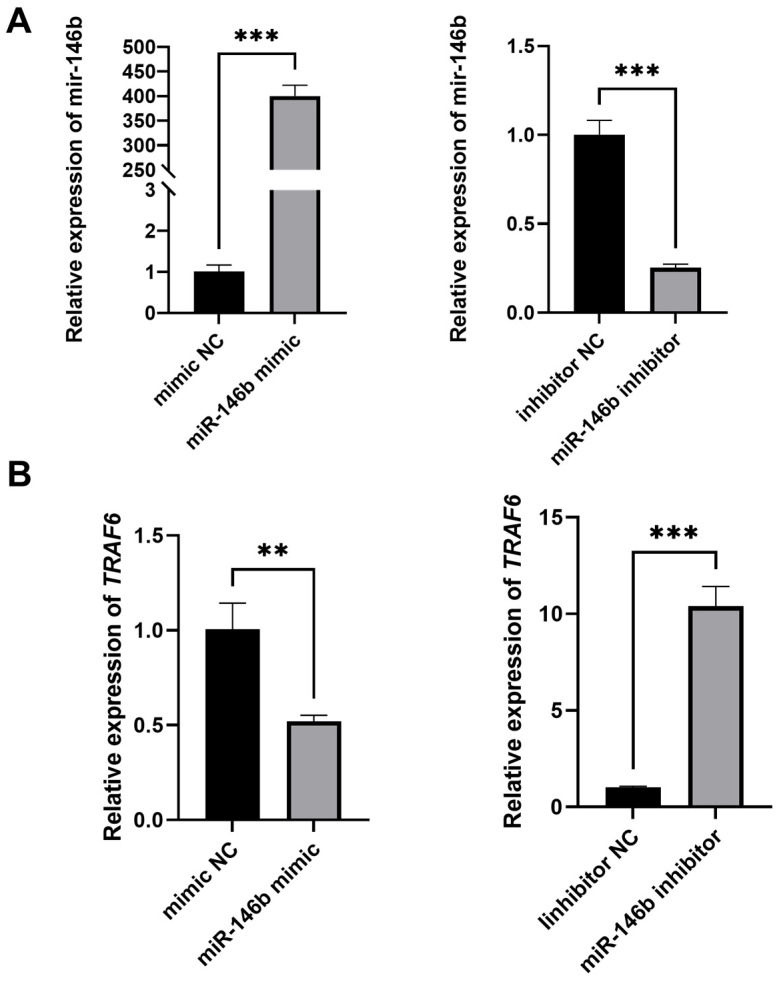
Effect of miR-146b on *TRAF6* expression in HEK293T cells. (**A**) The influence of miR-146b mimic and inhibitor on the mRNA expression level of miR-146b in HEK293T cells. (**B**) The impact of miR-146b mimic and inhibitor on the mRNA expression level of *TRAF6* in HEK293T cells. ** refers to *p* < 0.01, *** refers to *p* < 0.001.

To investigate the effect of miR-146b on *TRAF6* expression, HEK293T cells were transfected with either an miR-146b mimic or inhibitor. Transfection efficiency was confirmed by the increased level of miR-146b in the mimic group and the decreased level in the inhibitor group (Figure 4A). Compared with the mimic NC group, *TRAF6* mRNA levels were significantly reduced in cells overexpressing miR-146b. Conversely, inhibition of miR-146b led to an increase in *TRAF6* mRNA levels relative to the inhibitor NC group (Figure 4B). These results indicate a negative correlation between miR-146b and *TRAF6*, suggesting that *TRAF6* is a potential target gene of miR-146b in bovine mammary epithelial cells.

### 3.5. TRAF6 Expression Is Regulated by miR-146b Through 3′-UTR Binding

**Figure 5:**
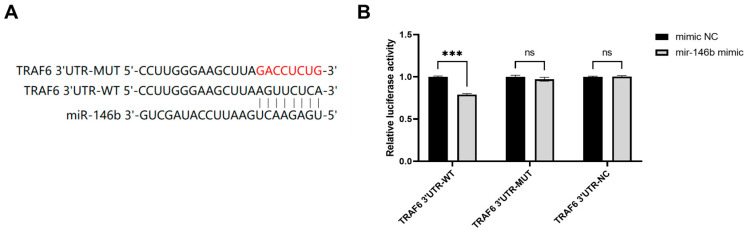
*TRAF6* is a molecular target of miR-146b. (**A**) TargetScan predicted the binding site of miR-146b to *TRAF6* 3′-UTR, where “GACCUCUG” is the mutated base. (**B**) The results of double luciferase activity detection. HEK293T cells were co-transfected with miR-146b mimic or negative control mimic (mimic NC) together with luciferase reporter vectors containing the wild-type (WT), mutant (MUT), or negative control (NC) 3′-UTR sequence of *TRAF6*. Luciferase activity was measured 48 h post-transfection. Relative luciferase activity was calculated as the ratio of Firefly luciferase activity to Renilla luciferase activity. *** refers to *p* < 0.001, ns refers to *p* > 0.05.

TargetScan analysis predicted that the 3′-untranslated region (3′-UTR) of *TRAF6* mRNA contains conserved binding sites for miR-146b (Figure 5A). To validate this prediction, a dual-luciferase reporter assay was performed in HEK293T cells. The results showed that co-transfection with the miR-146b mimic and the wild-type *TRAF6* (*TRAF6*-WT) reporter plasmid significantly reduced luciferase activity compared to co-transfection with the mutant *TRAF6* (*TRAF6*-MUT) reporter plasmid or the negative control (Figure 5B). These findings demonstrate that miR-146b can directly bind to the 3′-UTR of *TRAF6* and suppress its expression at the post-transcriptional level.

### 3.6. miR-146b Negatively Regulates TRAF6 mRNA and Protein Expression in LTA-Induced MAC-T Cells

**Figure 6:**
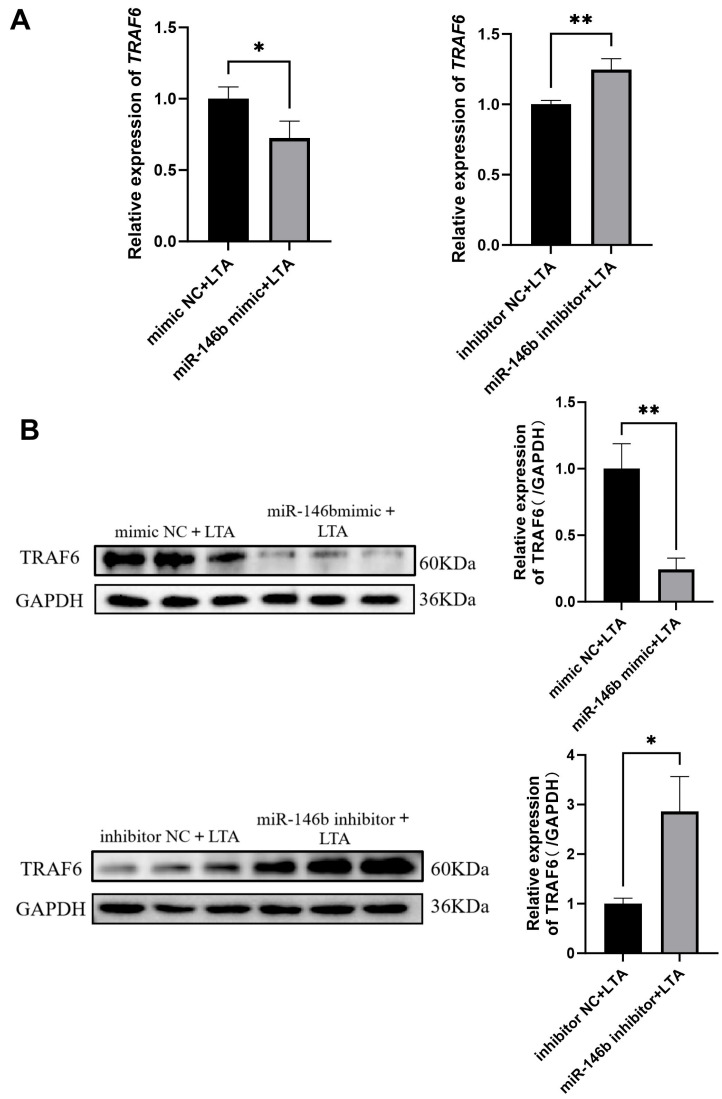
Effect of miR-146b on *TRAF6* expression in LTA-Induced MAC-T Cells. (**A**) mRNA expression level of *TRAF6* in MAC-T cells induced by LTA. (**B**) Protein expression level of TRAF6 in MAC-T cells induced by LTA. * refers to *p* < 0.05, ** refers to *p* < 0.01.

To further validate the regulatory role of miR-146b on *TRAF6* in LTA-induced MAC-T cells, we assessed *TRAF6* expression following miR-146b overexpression or knockdown using qRT-PCR and Western blot. The results demonstrated that miR-146b negatively regulates both the mRNA and protein levels of TRAF6 in inflammatory MAC-T cells (Figure 6A,B). Consistent with the dual-luciferase reporter assay results, we confirmed that *TRAF6* is a direct molecular target of miR-146b in MAC-T cells.

### 3.7. miR-146b Inhibits the Expression of Inflammatory Factors in LTA-Induced MAC-T Cells

**Figure 7:**
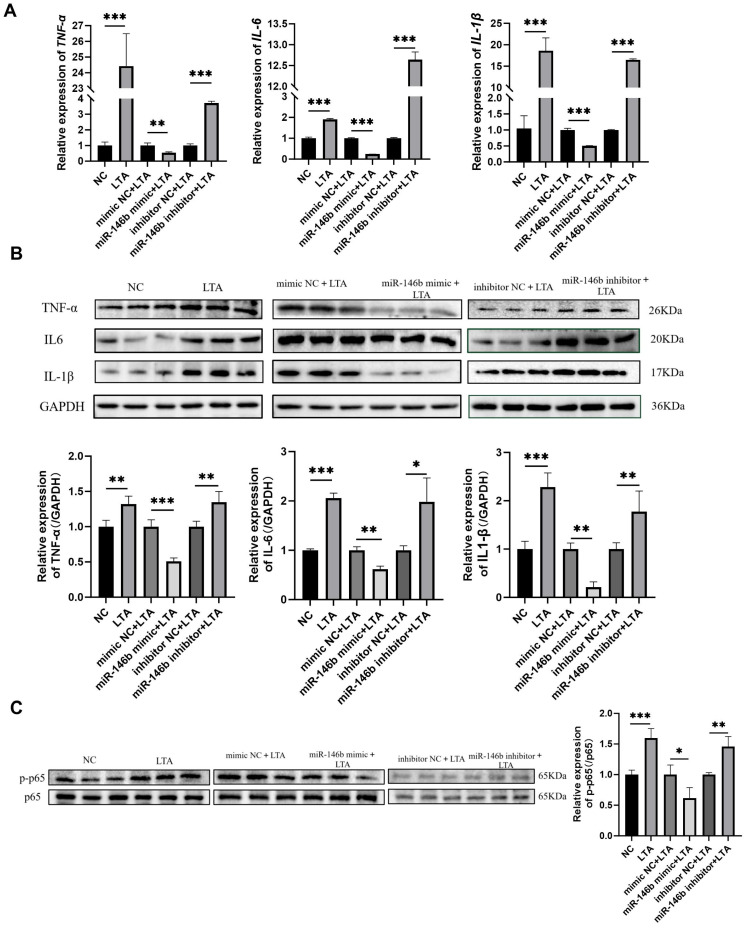
Regulatory role of miR-146b on TNF-α, IL-6, IL-1β, and NF-κB in LTA-induced MAC-T cells. (**A**) Effects of miR-146b overexpression or inhibition on the mRNA levels of inflammatory factors in LTA-induced MAC-T cells were detected by RT-qPCR. (**B**) Effects of miR-146b overexpression or inhibition on the protein levels of inflammatory factors in LTA-induced MAC-T cells were detected by Western blot. (**C**) Regulatory effects of miR-146b overexpression or inhibition on NF-κB in LTA-induced MAC-T cells were detected by Western blot. * refers to *p* < 0.05, ** refers to *p* < 0.01, *** refers to *p* < 0.001.

RT-qPCR analysis demonstrated that in the LTA-induced MAC-T inflammatory model, the mRNA expression levels of the inflammatory factors *TNF-α*, *IL-6*, and *IL-1β* were significantly elevated compared to the NC group. Conversely, transfection with an miR-146b mimic reduced the mRNA levels of *TNF-α*, *IL-6*, and *IL-1β*, whereas the miR-146b inhibitor increased their expression (Figure 7A). Furthermore, Western blot analysis revealed a consistent trend at the protein level (Figure 7B). Moreover, overexpression of miR-146b suppressed the expression of phosphorylated p65 (p-p65), while inhibition of miR-146b enhanced p-p65 levels (Figure 7C). These results indicate that miR-146b attenuates the expression and activity of phosphorylated NF-κB (pNF-κB), thereby downregulating the intracellular expression of the inflammatory factors TNF-α, IL-6, and IL-1β.

## 4. Discussion

Mastitis is an inflammatory process that induces pathological changes in the mammary tissue of dairy cows and leads to the impairment of bovine mammary epithelial cells [23]. While many therapeutic strategies have improved in recent years, the incidence rate of bovine mastitis remains at a high level. Because of the absence of effective treatment, infected cows are usually culled, leading to great economic losses for farmers [23]. Thus, investigating the mechanisms underlying inflammatory injury in bovine mastitis is necessary for developing strategies that will reduce its incidence. *Staphylococcus aureus* is considered one of the most important pathogens responsible for bovine mastitis. LTA is the major component of the Gram-positive bacterial cell wall, is capable of triggering immune signaling pathways in host cells [3]. This activation leads to the occurrence of oxidative stress, autophagy, and apoptosis in mammary epithelial cells. These properties make LTA an ideal agent for simulating inflammation caused by Gram-positive bacteria [24,25]. Thus, we established an inflammatory model with LTA in MAC-T cells in order to further study the pathogenesis process of mastitis and find possible strategies for preventing and treating mastitis.

Changes in the miRNA expression are one of the early cellular responses to extracellular stimuli. It has been reported that miRNAs may directly target pro-inflammatory cytokines and genes of the crucial signaling pathway, for example, JAK/STAT and MAPK signaling [26,27]. As one important member of the inflammation-associated miRNA family, the function of miR-146b in regulating the TLR/NF-κB signaling pathway has been confirmed in humans, mice, and other species [28]. However, its specific role in bovine mammary epithelial cells remains to be determined. In the current study, miR-146b expression was found to be significantly up-regulated in inflammatory MAC-T cells. These results indicated that miR-146b may participate in the development of bovine mastitis. Further overexpression of miR-146b resulted in a decrease in *TRAF6* expression, while inhibition of miR-146b promoted the expression of *TRAF6*. This suggests that *TRAF6* is a putative target gene of miR-146b. Therefore, we further conducted dual-luciferase reporter assays and proved that *TRAF6* is a direct molecular target of miR-146b. Additionally, this result was consistent with several studies conducted on other species or cell types. For example, several studies have shown that miR-146b inhibits inflammatory responses through Interleukin 1 Receptor Associated Kinase 1 (*IRAK1*) and *TRAF6* in human and murine immune cells [29,30]. Overexpression of miR-146b notably inhibited the expression of pro-inflammatory cytokines (TNF-α, IL-6, and IL-1β) in LTA-stimulated MAC-T cells, whereas inhibiting miR-146b showed the opposite effect. These findings suggest that miR-146b exerts its anti-inflammation effect through the downregulation of *TRAF6*. In addition, we found that miR-146b also affected the phosphorylation of NF-κB p65, and we speculated that, mir-146b inhibited the activation of the NF-κB pathway. CCK-8 assays showed that miR-146b promotes the viability of cells in the LTA-induced inflammatory model. It hinted that miR-146b could not only alleviate inflammation but also protect the injury of mammary epithelial cells mediated by LTA [31,32].

It is important to note that a specific “synchronous upregulation” expression pattern of miR-146b and TRAF6 was displayed in MAC-T cells during inflammation. This can be explained mainly by the temporal control of inflammatory signaling and peculiar features of miRNA-mediated post-transcriptional regulation. Mechanistically, binding of LTA to *TLR2* triggers the activation of a downstream MyD88-dependent pathway, which leads to recruitment and activation of TRAF6 [33]. Because *TRAF6* is a key adaptor protein in the NF-κB signaling pathway and also becomes a target of transcriptional regulation by NF-κB [34], inflammatory activation thus initiates a positive feedback loop: NF-κB nuclear translocation promotes the transcription of proinflammatory genes, including *TRAF6*, *IL-6*, and *TNF-α* [35], causing a rapid transcriptional induction of *TRAF6*. Meanwhile, a negative feedback mechanism was elicited to maintain immune homeostasis. In the early to intermediate stage of inflammation, the rate and strength of NF-κB-mediated *TRAF6* transcription may overcome the suppressive activity of miR-146b, which was also induced by NF-κB. Consequently, both exhibited an overall up-regulation at the population level. Of note, the induction of miR-146b should not be misinterpreted as proinflammatory. It represents an adaptive regulatory response. Once the level of miR-146b increases to a functional threshold, it suppresses the expression of *TRAF6*, resulting in restraint of NF-κB signaling, which limits inflammatory amplification and protects mammary epithelial cells from injury.

In the present study, our findings link bovine mastitis to miR-146b; its overexpression in LTA-challenged MAC-T cells curbs inflammatory responses while enhancing cell viability, and this protection is mediated through direct targeting of *TRAF6* to suppress the inflammatory cascade.

## 5. Conclusions

In conclusion, this study demonstrates that miR-146b plays a critical anti-inflammatory role in LTA-stimulated bovine mammary epithelial cells by targeting *TRAF6*. Our findings show that miR-146b binds with high specificity to the seed sequence in the 3′-untranslated region (3′-UTR) of *TRAF6* mRNA, leading to the repression of *TRAF6* translation and subsequent reduction in protein levels, with a significant reduction in the expression of its downstream proinflammatory cytokines. Overexpression of miR-146b enhances cell viability while markedly reducing inflammatory responses, highlighting its potential as a therapeutic target for mastitis.

## Figures and Tables

**Table 1:** Fluorescence quantitative primer sequence.

Name	Primer Sequence	Size of Amplification Fragment
miR-146b	F:ACGGCACTGAGAACTGAATTCCA	65 bp
	R:ATCCAGTGCAGGGTCCGAGG	
*TNF-α*	F:ATGTGGAGCTGGCGGAGGAG	93 bp
	R:GGAGGAAGGAGAAGAGGCTGAGG	
*IL-6*	F:CCTCTCTGGCAAGAGACTTCCAT	86 bp
	R:AGTCTCCTCTCCGGACTTGT	
*IL-1*β	F:ATGAAGAGCTGCATCCAACACCTG	147 bp
	R:ACCGACACCACCTGCCTGAAG	
*TRAF6*	F:TGGAACTGAGGCATCTTGAGGAG	82 bp
	R:TGGAAGGGACGCTGGCATTG	
*U6*	F:CGCTTCACGAATTTGCGTGTCAT	89 bp
	R:GCTTCGGCAGCACATATACTAAAAT	
*GAPDH*	F:CGCATCCCTGAGACAAGATGG	207 bp
	R:TCCCGTTCTCTGCCTTGACT	

## Data Availability

Data are available upon reasonable request.
